# MXene synthesis in a semi-continuous 3D-printed PVDF flow reactor†

**DOI:** 10.1039/d4na00991f

**Published:** 2025-03-19

**Authors:** Molly J. Clark, Alice E. Oakley, Nikolay Zhelev, Marina Carravetta, Thomas Byrne, Adrian M. Nightingale, Nuno Bimbo

**Affiliations:** a Mechanical Engineering Department, School of Engineering, Highfield Campus, University of Southampton Southampton SO17 1BJ UK a.nightingale@soton.ac.uk; b School of Chemistry and Chemical Engineering, Highfield Campus, University of Southampton Southampton SO17 1BJ UK n.bimbo@soton.ac.uk; c Centre of Excellence for Continuous Digital Chemical Engineering Science, Faculty of Engineering and Physical Sciences, University of Southampton Southampton SO17 1BJ UK

## Abstract

Two-dimensional transition metal carbides, nitrides and carbonitrides known as MXenes represent a promising class of functional materials for electrochemical energy storage, catalysis, electromagnetic shielding, and optoelectronics. Typical synthesis methods require highly concentrated acids and HF-containing or HF-forming chemicals, under batch conditions. Environmentally friendly, safe, efficient, and scalable synthesis methods for MXenes have been identified as the number one research challenge for MXene research over the next decade. Here we use flow chemistry to present a semi-continuous synthesis of Ti_3_C_2_T_*z*_ in a custom 3D-printed reactor. The synthesis is safer and is the first step towards scalable methods, yielding fully etched MXenes with better removal of Al from the starting MAX phase compared to the equivalent batch procedure.

## Abstract 2

Etching of MXene Ti_3_C_2_T*_z_* is done for the first time using semi-continuous methods in a custom 3D-printed reactor.

## Graphical abstract

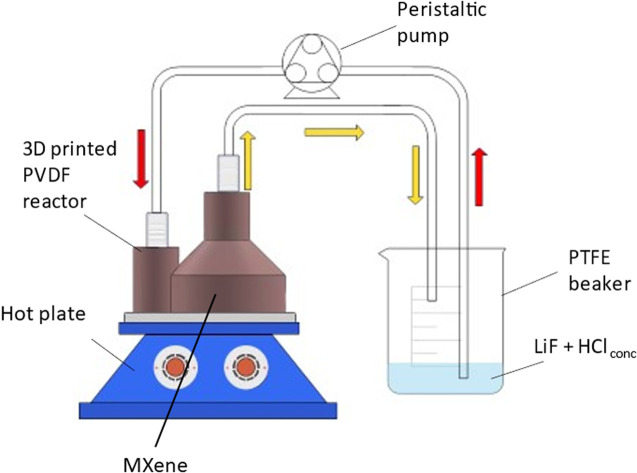

MXenes are a new family of two-dimensional materials made of carbides, nitrides, and carbonitrides of transition metals. They have shown great promise in many applications, including electrochemical energy storage, catalysis, electromagnetic shielding, and optoelectronics, as they have many desirable features such as high electrical conductivity, robust mechanical properties, and efficient absorption of electromagnetic waves.^1^ MXenes are obtained from precursor MAX phases, (M = early transition metal, A = a group 13 or 14 element, and X = C or N), through the selective etching of the A group layers, which give nanosheet MXene structures with the general formula M_*n*+1_X_*n*_T_*z*_ (*n* = 1–3, T_*z*_ = surface termination).^2^ Etching is typically achieved using highly caustic fluoride-containing solutions such as hydrofluoric acid, hydrochloric acid and lithium fluoride mixtures, or ammonium bifluoride.^3^ However, the use of fluoride-based solutions is highly hazardous, requiring chemically inert process equipment and care when handling. Consequently, scale up is problematic^4^ and if MXenes are to be used in a wide range of applications as predicted, industry-scale manufacturing strategies must be found.^5^ Recently, scalable synthetic methods that are environmentally friendly, safe, efficient, and scalable were identified as the number one challenge for MXene research over the next decade.^6^

MXenes have been synthesised using fluoride-free methods,^7–9^ and other methods, such as electrochemical etching^10–13^ and synthesis in molten salts^14^ have also been used. Different synthetic methods significantly affect the resulting product, as the composition of the surface functional groups, which highly influence MXene properties, differs depending on the method used. Recent methods based on molten salts, for example, can be tailored to give imido-, sulfur-, chlorine-, selenium-, bromine-, and tellurium-rich surfaces.^15^ There have also been recent reports of bottom-up synthesis strategies involving chemical vapour deposition^16^ which produced MXenes previously unattainable from MAX phase precursors. Despite the breadth of available methods, so far to our knowledge all synthesis strategies have been done in batch and, despite some reports of scale-up such as the one by Shuck *et al.*^17^ (where a large reactor was used to synthesise 50 g of MXene), no attempts have been reported of MXene etching using continuous methods.

Continuous processing has many advantages over batch synthesis, such as smaller equipment and space requirements, smaller reactor volumes and hence increased safety and space-time yields, lower variation of product quality, simpler process monitoring and control, easier scale-up, and higher productivity.^18^ So far, using continuous methods in MXene synthesis has proven elusive, due to harsh operating conditions (HF or HF-forming chemicals) very long reaction times (24 or 48 hours typically for the synthesis of the most researched MXene, Ti_3_C_2_T_*z*_), and handling of solids, which are significant hurdles in the transition from batch to continuous. To address this, we took a hybrid semi-continuous approach whereby etchant was continuously flowed through a volume containing a constantly agitated suspension of MAX phase, meaning that only 50 ml of etchant were used in the process (typical batch production methods use similar volumes). While this does not allow continuous production of MXene, the continuous flow of etchant means that HF could be generated inline (by combining acid and fluoride sources) and used etchant could be autonomously neutralised inline, reducing manual handling risks. To make the flow reactor (Fig. 1A and B) we used fused deposition modelling (FDM) 3D printing. FDM printing creates parts by extruding thermoplastic through a heated nozzle on a 2D plane to build up the 3D model layer-by-layer. One of the key advantages of FDM over other 3D printing methods is the range of available materials. Here we took advantage of this by using a highly inert fluoropolymer, polyvinylidene difluoride (PVDF), compatible with HF-based etchants. While there are many previous reports of 3D printed reactor vessels, this is the first use (to our knowledge) of PVDF to allow use of such aggressive reagents. A discussion of the printing considerations when using PVDF is given in ESI.† A key advantage of FDM is that pre-programmed pauses can be added during printing (the “print–pause–print”, PPP, approach) to incorporate external materials such as membrane filters,^19^ stirrer bars,^20^ and electromagnets.^21^ Here we use this to incorporate a PVDF filter membrane to ensure the MAX phase remains within the reactor and is not carried out by the etchant flow, as well as an encapsulated stirrer bar to ensure constant agitation (Fig. 1C).

**Fig. 1:**
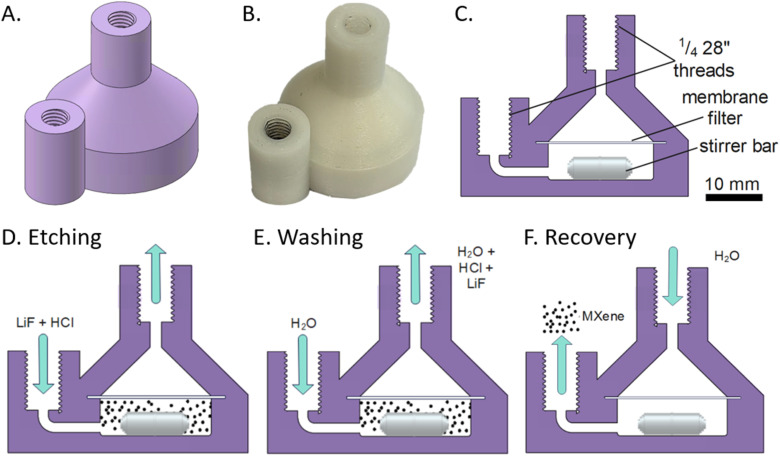
(A) CAD image of the reactor. (B) Corresponding image once fabricated. (C) Cross section showing internal geometry and parts. (D)–(F) Cartoons showing how the reactor was used for etching (D), neutralisation (E) and MXene recovery (F).

The MAX phase (1 g) was made into a suspension and then loaded into the reactor using a syringe. Etchant was then continuously supplied over 48 hours (Fig. 1D) using a peristaltic pump, followed by neutralisation *via* elution (Fig. 1E and S1 in ESI† for plot of pH *vs.* time) and then recovery by backflowing with water (Fig. 1F). For the continuous sample, a yield of 671 mg (66.9%) was retrieved from the device after neutralisation, with material likely lost during initial loading of the MAX phase into the device and when filtering the retrieved slurry post-etching. Typical batch methods have a >90% yield so improvements on product recovery are needed to reach similar values. Areas to investigate for increased product recovery include improvements on washing and filtration, and reactor design optimisation. In addition, due to the nanostructure of MXenes, it is possible some smaller MXene particles may have passed through the membrane filter. For comparison, an equivalent batch synthesis was performed using the same quantities and concentrations of reagents, the same reaction time, but continuously stirred in a PTFE beaker, and then neutralised by repeated centrifugation, supernatant removal, and resuspension steps.

Powder X-ray diffraction (PXRD) results (Fig. 2A) confirmed that both methods successfully produced crystalline Ti_3_C_2_T_*z*_ with the diffraction pattern matching those we previously reported^22–24^ and the characteristic MAX phase (002) peak at 9.5° being almost imperceptible, indicating almost complete removal of the starting material. The higher angle MAX phase peaks are still present, albeit significantly reduced in size, indicating some left-over or unetched MAX phase. These peaks are notably smaller for the continuous method compared to the batch method. Other impurities such as LiF are not present as indicated by the absence of responses at 39°, showing that the neutralisation and washing steps were successful. Comparing the differences between the two MXene products, the flow sample shows slightly more well-defined peaks compared to the batch sample, most notably the (002) peak located between 5 and 6° (Fig. 2B). This peak shows a peak position difference of 0.58° for the two methods – indicative of different interlayer spacings for the 2D nanosheets structures, and probably caused by the re-arrangement of hydrogen-bonded guests such as water molecules between the layers.^25^ This might be due to differences in the etching procedure or different drying of the sample.

**Fig. 2:**
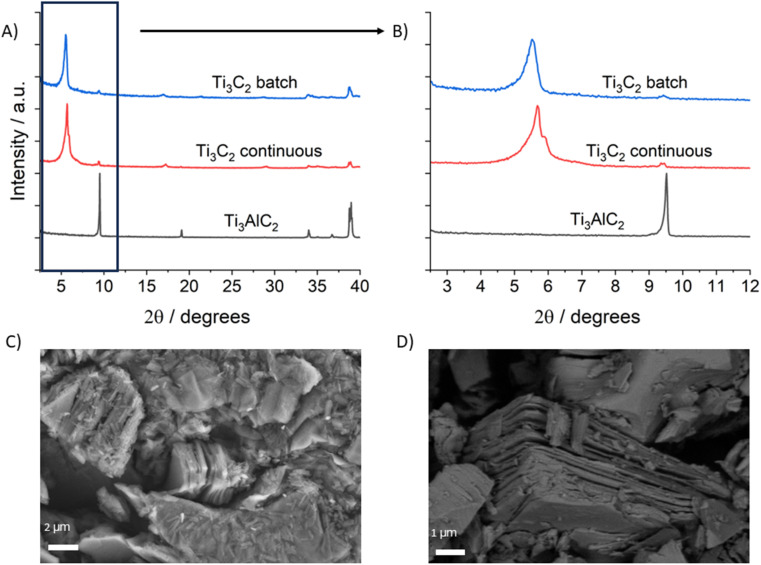
Powder X-ray diffractograms for the precursor MAX phase, the batch sample, and the continuous sample: (A) shows 2*θ* angles up to 40°, while (B) shows the 2*θ* angles up to 12°. (C) and (D) Show SEM images for the batch and continuous samples, respectively. Both images display the typical layered morphology of etched Ti_3_C_2_T_*z*_.

Scanning electron microscopy (SEM) of the products (Fig. 2C and D) showed successful exfoliation of the MAX phase, with both samples showing accordion-like structures with clear separation of individual layers, matching those seen in literature.^3^ Elemental analysis *via* energy dispersive X-ray spectroscopy (EDS, detail in ESI†) showed low atomic weight of Al for both samples, consistent with successful conversion from the starting MAX phase. The Al content was notably lower for the continuous sample (0.21%) compared to the batch sample (3.86%) however, indicating that better exfoliation and/or washing had occurred, and consistent with observations of the magnitude of the higher-angle XRD peaks (Fig. 2A). These results were further confirmed by X-ray photoelectron spectroscopy (XPS) and solid-state NMR.

The XPS results confirmed that Al remained in the batch-produced samples, but not in the flow sample. Surface oxidation was evident in both cases, likely due to the long (unoptimised) etching time for both samples.^26^ Water was also present, either due to intercalated water (difficult to remove from the sample), or adsorbed water on the surface after exposure to ambient conditions. Otherwise, the results (shown in ESI†) were similar for both samples, consistent with successful etching of the MAX phase to produce MXenes.

We used ^27^Al and ^1^H solid-state NMR to further characterise the MXene surface.^27^^27^Al solid-state NMR results (Fig. 3A) confirmed EDS and XPS results, with no Al present in the flow-produced sample, but some Al present in the batch sample. The ^1^H spectra shown in Fig. 3B and the corresponding fits (data shown in ESI in Tables S5 and S6†) show that the spectra is dominated by peaks at 5.13 ppm (for the batch sample) and 4.66 ppm (for the continuous sample), indicative of water^24,27^ and consistent with XPS results. The absence of significant peaks above 10 ppm suggest a small proportion of –OH surface functional groups in both samples, consistent with previous results.^28,29^ TEM measurements were done on the continuous and batch samples (Fig. S9 in ESI†), which show that both sets of samples exhibit similar morphologies. UV-vis spectroscopy was also carried out in the samples, and is included in ESI (Fig. S10).†

**Fig. 3:**
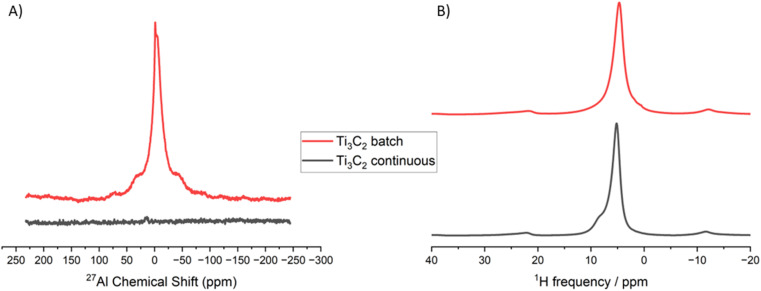
^27^Al and ^1^H solid state NMR solid-state NMR on the batch and continuous Ti_3_C_2_T_*z*_ MXene samples. (A) is the ^27^Al NMR spectra for the batch and continuous samples, data is adjusted by sample weight. (B) ^1^H NMR spectrum for the batch and continuous samples.

The multiple tools used to characterise the batch and continuous samples show that, for both cases, Ti_3_AlC_2_ has been successfully etched to yield crystalline Ti_3_C_2_T_*z*_ with morphology and surface properties in keeping with previous literature reports. While all analyses show that no Al is present in the flow-etched sample, which indicates successful etching and washing of the sample, the standard batch sample still contained small levels of Al due to either incomplete etching and/or washing. The reason for this is unknown at present but may be due to better mixing in the printed reactor, something that could be further optimised in subsequent designs – taking advantage of the versatility of 3D printing to create almost arbitrary structures. Other areas to investigate in reactor design are related to optimisation for safer operation, including less handling of chemicals and easier neutralisation and removal protocols.

The successful etching of Ti_3_C_2_T_*z*_ presented in the above results is, to our knowledge, the first obtained using flow methods. It should be noted that there was much less manual handling of the etchant, in particular during the neutralisation stage, which makes for an overall safer production method. Also, the washing only uses 75.2 ml of deionised water, as opposed to batch methods in which centrifuging cycles mean around 200 ml of water are used for the same amount of product. Here the etchant was recycled for ease, however safety of the setup could be enhanced by formulating the etchant inline, by introducing the LiF and HCl separately so that HF is only generated in the reactor, and immediately neutralising the etchant inline after contacting with the MAX phase.

In this report we demonstrate for the first time the semi-continuous etching of a MAX phase (Ti_3_AlC_3_) using a bespoke device produced *via* 3D printing. To our knowledge, this is also the first report in which PVDF has been used in 3D-printed reaction vessels, and hence can be used for highly aggressive reagents such as the HF-containing etchant used here. Our continuous approach reduces manual intervention and risk associated with typical MXene synthesis, enables easier neutralisation, outputs a product with less remaining Al and allows for easier standardisation of the synthesis. However, improvements must be done on product recovery, as lower yields were obtained than typical batch synthesis. As the reactor is 3D printed it can be easily edited to optimise mixing and incorporate other functionalities (extra inlets/outlets), giving it the potential to scale the reaction for incorporation into larger processes. Due to the importance of scaling up MXene production, we hope our study can stimulate further work in the area, especially enabling a greater understanding of the continuous etching process, and how it influences etching kinetics, and the chemical and morphological composition of the products. Future work in this area should focus on optimising the reactor design, improving yield and product recovery, coupling reaction monitoring tools to optimise the process, and investigating methodologies for scaling up production of bigger quantities of MXenes.

## Data availability

The data supporting this article have been included as part of the ESI.†

## Conflicts of interest

There are no conflicts to declare.

## Supplementary Material

NA-OLF-D4NA00991F-s001
