# Supplementary material for: MXene synthesis in a semi-continuous 3D-printed PVDF flow reactor
Source: Nanoscale Adv. 2025 Mar 19;7(8):2166–70. doi: 10.1039/d4na00991f (PMC11934147; doi:10.1039/d4na00991f)
Supplement: NA-007-D4NA00991F-s001 [file NA-007-D4NA00991F-s001.pdf]

Supplementary Information (SI) for Nanoscale Advances.
This journal is © The Royal Society of Chemistry 2025

Supporting Information

MXene synthesis in a semi-continuous 3D-printed PVDF flow reactor

Molly Clark[a,b,c], Alice Oakley[b], Nikolay Zhelev[b], Marina Carravetta[b], Thomas Byrne,[b] Adrian Nightingale[a,c] and Nuno Bimbo[b,c]

[a] *Mechanical Engineering Department, School of Engineering, Highfield Campus, University of Southampton, Southampton, SO17 1BJ, UK*

[b] *School of Chemistry and Chemical Engineering, Highfield Campus, University of Southampton, Southampton, SO17 1BJ, UK*

[c] *Centre of Excellence for Continuous Digital Chemical Engineering Science, Faculty of Engineering and Physical Sciences, University of Southampton, Southampton, SO17 1BJ, UK*

EXPERIMENTAL METHODS

Materials

Polyvinylidene difluoride (PVDF) filament (2.85 mm diameter, “FluorX”) was obtained from 3DXTECH. PVDF filter membranes (Ø= 47 mm) were cut into smaller diameters (Ø= 23.5 mm). The precursor MAX phase (Ti_3AlC_3) was purchased from Laizhou Kai Kai Ceramic Materials Co., Ltd. Hydrochloric acid (HCl, 12 M) was purchased from Fisher and lithium fluoride was purchased from Sigma Aldrich.

Device design and fabrication

The device was designed using Solidworks (Dassault software) and exported (.3mf format) into Cura (Ultimaker) to generate a .gcode file for printing. The device included threaded inlet/outlet recesses to allow watertight connection using ETFE ¼”-28 flangeless fittings (Idex, Cole Parmer). All internal channels had a diameter of 1 mm. The design featured an open cylindrical chamber with a recess to support the membrane filter, at a height of 200 µm and a diameter of 23.7 mm (200 µm larger than the membrane diameter). Incorporation of the membrane created a contained chamber (h= 6.97 mm, Ø= 19.2 mm, V= 1.98 mL) below the membrane, where the MAX phase is loaded during etching, as shown in Figure 1.

The device was printed in PVDF using an Ultimaker 3, with an Ultimaker adhesion sheet applied to the print bed. Print parameters were adapted from previous reports and were set as follows: layer height – 200 µm; infill - 60%; print speed – 20 mm/s; flow rate - 100%; fan speed - 0%; wall line count - 3; scaling - 115% (to account for shrinkage of the filament upon cooling); nozzle temperature – 260 °C. The print was paused after the membrane recess was formed to add the stirrer bar and the external membrane before resuming the print and sealing the membrane in place. To ensure the membrane didn’t move upon resumption, a small amount of all-purpose adhesive (UHU) was applied around the circumference.

It should be noted that the printing parameters differ appreciably from our previous report of printing devices in polypropylene [1] and other previous reports of 3D printed fluidics [2] and is due to the large degree of shrinkage exhibited by the PVDF as it cooled, and the difference in the membrane material. Firstly, the design was scaled up to 115% of nominal size in the Cura software to counteract the shrinking and ensure the final device matched nominal design dimensions. Issues with buildplate adhesion were alleviated by reducing the infill density to 60%. Finally, we note that in previous work incorporating membranes in polypropylene devices we increased the filament flow rate for the first layer after the membrane was inserted to ensure a good conformal bond between membrane and print. Here this was unnecessary - either due to the shrinking, or more likely due to the molten printed PVDF remelting the membrane on contact, itself made of PVDF.

Standard MXene batch synthesis and neutralisation

The batch sample was obtained using a procedure we previously reported [3], which is adapted from Ghidiu et al. [4]. A solution of 6 M of HCl was diluted from a stock solution of 12 M HCl. This was placed on a PTFE 200 ml HF-resistant beaker on a hot plate, which was warmed to 40 °C. A mass of 1.0 g of LiF was weighed and slowly added to the HCl solution. After 5 min, 1.0 g of Ti_3AlC_2 was weighed and slowly added to the HCl and LiF solution. The solution was stirred with a magnetic stirrer for 48 hours with the hotplate set at 40 °C, after which the product was isolated: The mixture was decanted to centrifuge tubes, mixed with deionised water in an approximate 1:1 volume ratio and centrifuged at 5,000 rpm for 10 mins. The supernatant was removed, and the resulting sediment redispersed with vortexing in deionised water. The procedure was repeated three times after which the pH was confirmed to be close to neutral using indicator paper. The resulting sediment was then collected and left to dry in a fumehood for 24 hours.

MXene synthesis and neutralisation in 3D printed flow reactor.

The MAX phase (Ti_3AlC_3) (1.02 g) was loaded into the device as a slurry in DI water using a syringe. A 1:1 solution of lithium fluoride and (12 M) hydrochloric acid was prepared (50 mL) and contained within a PTFE beaker. The etchant solution was supplied to the device using a peristaltic pump (Ismatec Reglo ISM834) fitted with Viton fluoroelastomer tubing (WZ-97629-18 Ismatec Pump Tubing, 3-Stop, Viton, 0.51 mm ID). PTFE tubing was inserted into the Viton pump tubing to take the etchant to the device, being attached to the device using ETFE ¼”-28 flangeless fittings. Similarly, the etchant exited the device via PTFE tubing attached with ¼”-28 flangeless fittings and was carried back to the etchant reservoir. To ensure a leak-free seal, all fittings to the device featured gaskets made by punching Viton sheet. The etching solution was continuously flowed through the device for 48 hours at a flow rate of 114 µL/min under stirring. A diagram of the setup can be found in Figure 1.

Following the 48 hr etching procedure of the continuous sample, neutralisation with water is required to remove the etchant and wash the product. The etching solution beaker was removed and replaced with a beaker of DI water to be flowed through the device and a second empty beaker for collection at the outlet. The pH of the eluent leaving the device was monitored using universal indicator paper and neutralisation was established after 11 hrs. To collect the product the flow direction was reversed, as shown in Fig. 1F. The product was filtered and dried overnight.

Characterisation of samples

<u>XRD</u>

The scanning electron microscopy characterisation involved imaging the sample morphology using a Carl Zeiss Sigma 500 VP field emission electron microscope (FE-SEM), coupled with an Oxford Instruments Ultim Max 170 mm2 energy dispersive X-ray spectroscopy detector (EDS) for studying the composition.

<u>XPS</u>

XPS Analysis was performed using a Thermo NEXSA XPS fitted with a monochromated Al kα X-ray source (1486.7 eV), a spherical sector analyser and 3 multichannel resistive plate with 128 channel delay line detectors. The instrument was calibrated to gold metal Au 4f (83.95 eV) and dispersion adjusted to give a binding energy of 932.6 eV for the Cu $2p_{3/2}$ line of metallic copper. The Ag $3d_{5/2}$-line FWHM at 10 eV pass energy was 0.56 eV. The source resolution for monochromatic Al Kα X-rays is ~0.3 eV. The instrumental resolution was determined to be 0.32 eV at 10 eV pass energy using the Fermi edge of the valence band for metallic silver. The resolution with charge compensation system on <1.5 eV FWHM on PTFE. All data was recorded at 72 W and X-ray beam size of 400 x 200 µm. Survey scans were recorded at a pass energy of 200 eV, and high-resolution scans recorded at a pass energy of 40 eV. Electronic charge neutralization was achieved using a dual-beam low-energy electron/ion source (Thermo Scientific FG-03). The ion gun current was equal to 150 µA and the ion gun voltage was equal to 45 V. All sample data was recorded at a pressure below 10^{-8} Torr and at a room temperature of 294 K.

NMR

Samples were packed into 2.5 mm zirconium oxide rotors with Vespel® caps and spectra were acquired at a spinning rate of 6 kHz for ^{27}Al spectra and 10 kHz for ^{1}H. ^{1}H and ^{27}Al spectra were acquired in double resonance mode on a Bruker Advance Neo 600 MHz wide bore spectrometer, using a 14.1 T field, in air. The chemical shift axes for the ^{27}Al and ^{1}H spectra were referenced using the octahedral site of yttrium aluminium oxide and adamantane as indirect reference, respectively. ^{27}Al spectra were acquired with 512 scans and a pulse delay of 10 seconds, ^{1}H spectra were acquired with 128 scans and a pulse delay of 4 seconds. The ^{1}H and ^{27}Al spectra for the flow and batch samples were normalised to the sample mass used which was 17.1 mg and 21.5 mg respectively. Fitting of the ^{1}H spectra was carried out using a dedicated MATLAB script.

TEM

Transmission electron microscopy imaging was done on a ThermoFisher Tecnai 12, with a Morada G2 camera, at 120 kV.

UV-Visible spectroscopy

Four solutions were prepared for UV-Vis spectroscopy, in which the MXene samples were mixed with water. There were two solutions each of the batch and continuous samples, with the following concentrations: Batch 1 has a concentration of 3.99 g/L, Batch 2 has a concentration of 2.00 g/L, Continuous 1 has a concentration of 4.00 g/L and Continuous 2 has a concentration of 2.00 g/L. The samples were sonicated for 3 minutes prior to UV-Vis measurements, which were done on a Perkin-Elmer Lambda 750S with integrating sphere in the range 200 to 700 nm. The baseline was automatically subtracted in the instrument by measuring a sample with water. All data was normalised.

SUPPLEMENTARY RESULTS

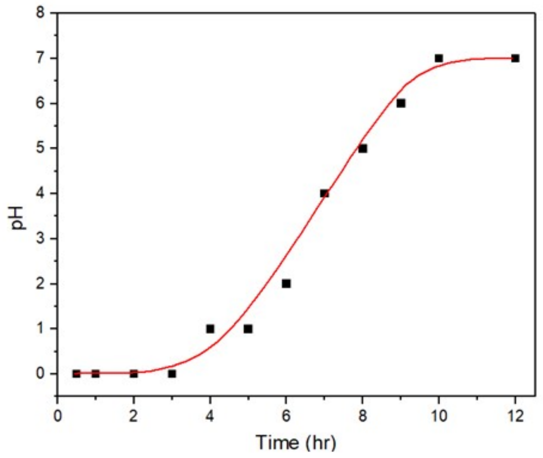

Figure S1: pH change over time for the neutralisation in the semi-continuous device.

EDS

EDS analysis was performed on both the continuous and batch samples. The results are shown in Figure S2 and Table S1 for the continuous sample and in Figure S3 and Table S2 for the batch sample. The data shows higher atomic % for C and O in the continuous sample, while the batch sample showed a higher atomic % of F. There are also traces of Ca present in the continuous sample (1.71% atomic) which were not present in the batch sample which can only have come from contamination due to human error at some point in the process.

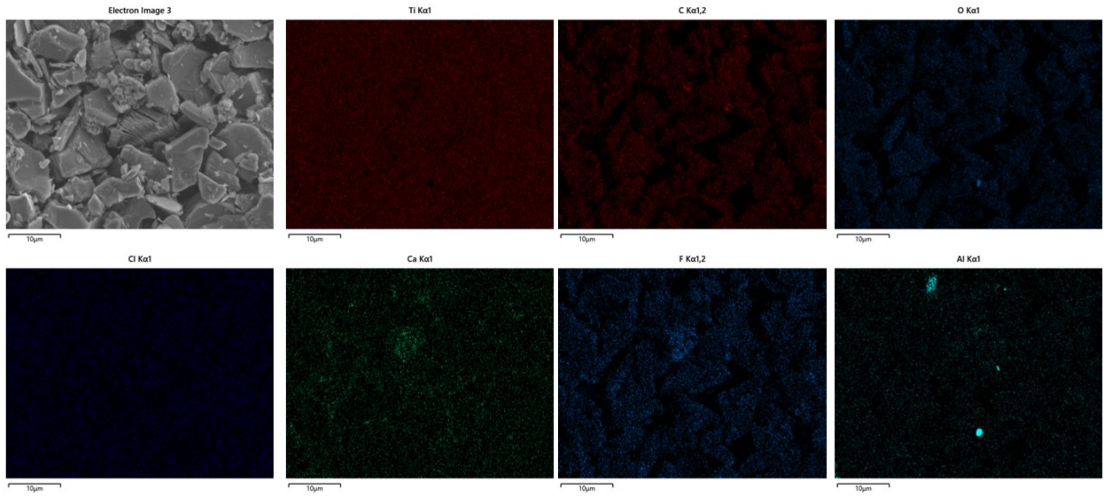

Figure S2 Energy dispersive X-ray spectroscopy (EDS) performed on the flow-produced sample. Detected elements include Ti, C, O (top row), Cl, Ca, F, Al (bottom row).

Table S1 Mapping of the EDS spectrum presented in Figure S2.

Element	Line Type	Apparent Concentration	Wt%	Wt% Sigma	Atomic %	Standard label
C	K series	15.96	11.09	0.09	25.16	C Vit
O	K series	26.17	14.66	0.15	24.96	SiO_2
F	K series	29.85	7.94	0.09	11.38	CaF_2
Al	K series	0.60	0.20	0.02	0.21	Al_2O_3
Cl	K series	6.34	2.20	0.03	1.69	NaCl
Ca	K series	7.88	2.52	0.04	1.71	Wollastonite
Ti	K series	143.69	61.39	0.16	34.91	Ti
Total			100.0		100.02	

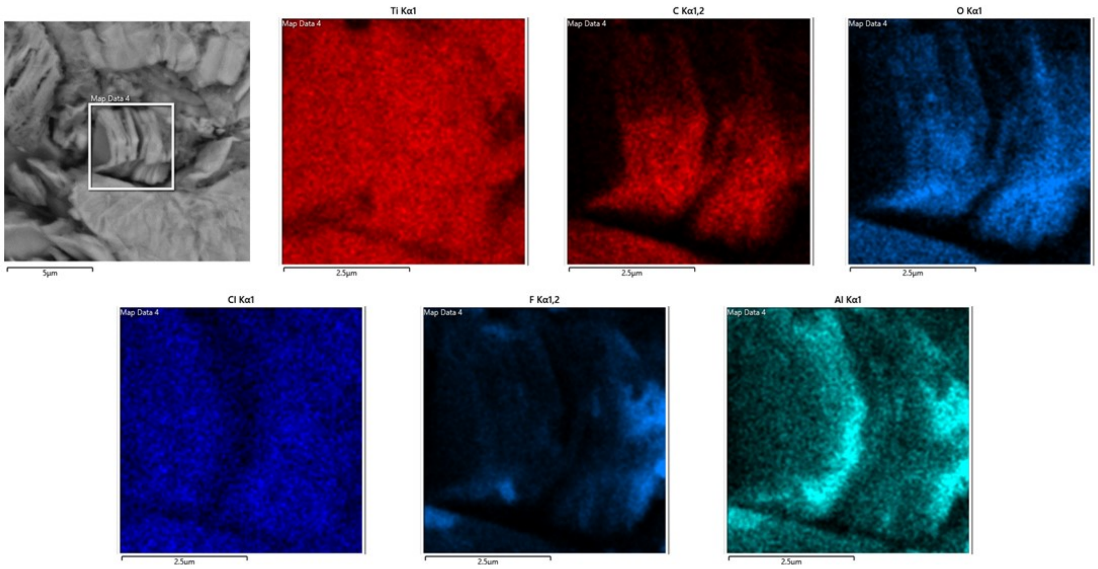

Figure S3 Energy dispersive X-ray spectroscopy (EDS) performed on the batch sample. Detected elements include Ti, C, O (top row), Cl, F, Al (bottom row).

Table S2 Mapping of the EDS spectrum presented in Figure S3.

Element	Line Type	Apparent Concentration	Wt%	Wt% Sigma	Atomic %	Standard label
C	K series	8.17	7.01	0.06	16.26	C Vit
O	K series	18.01	9.43	0.10	16.41	SiO_2
F	K series	76.43	18.40	0.08	26.98	CaF_2
Al	K series	10.80	3.74	0.03	3.86	Al_2O_3
Cl	K series	10.68	3.80	0.03	2.98	NaCl
Ti	K series	133.04	57.63	0.11	33.51	Ti
Total			100.01		100.0	

XPS

Another technique that has been routinely used to characterise MXenes is XPS. It has many advantages, including sensitivity to most elements (except H and He) and ability to characterise bonding environments and elemental compositions [5]. For MXenes, it has been widely used to characterise and quantify surface functional groups [5]. We have used XPS to analyse both the batch and continuous samples to understand the bonding and composition of the samples. We used CasaXPS version 2.3.25PR1.0 for the analysis [6] and the following guidelines, which includes most of the suggestions by Natu et al. [5]:

1. Tougaard background for all the analysed spectra.
2. All peaks were fitted with an asymmetric Lorentzian peak shape.
3. The bonding energies were calibrated using the 282.0 eV peak that corresponds to the C-Ti-C bonds.
4. The 2p spectra (Ti, Ca, Al, Cl) were fitted with two components to the $2p_{3/2}$ and $2p_{1/2}$ peaks. The area ratio between these peaks at $2p_{3/2}$ and the $2p_{1/2}$ was fixed at 2:1.
5. For the 2p spectra in the Ti sample, the separation ΔTi_{2p} from the Ti $2p_{3/2}$ and Ti $2p_{1/2}$ is kept constant for all components, and is equal to 6.1 eV for $Ti_3C_2T_z$-related peaks and 5.6 eV for oxides and oxyfluorides.
6. The full-width at half maximum (FWHM) is equal for the fits of components under the Ti $2p_{3/2}$ peak and for the fits of components under the Ti $2p_{1/2}$ peak.

The high-resolution XPS full spectra are presented in Figure S4 for the batch and continuous samples.

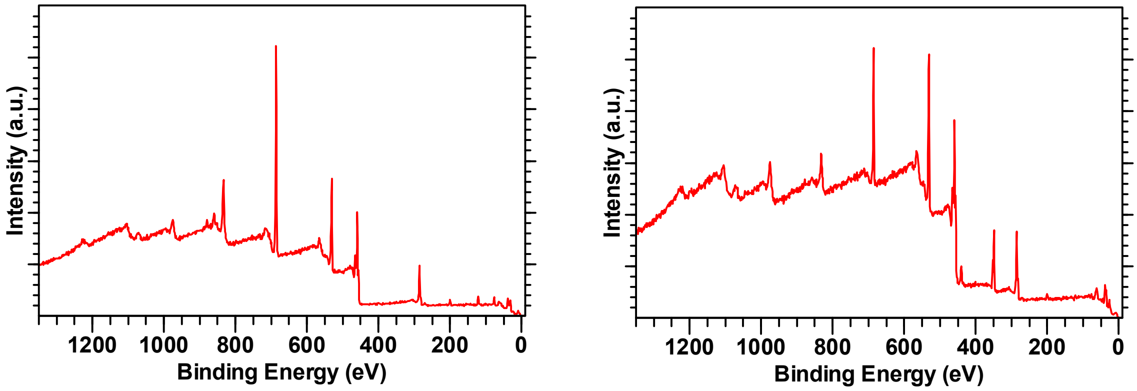

Figure S4 High-resolution XPS spectra for the batch (left-hand side) and continuous sample (right-hand side).

<u>Batch sample</u>

The XPS fittings for the O 1s, C 1s, Ti 2p and F 1s regions are shown below in Figure S5. The peak fitting data is displayed in Table S3.

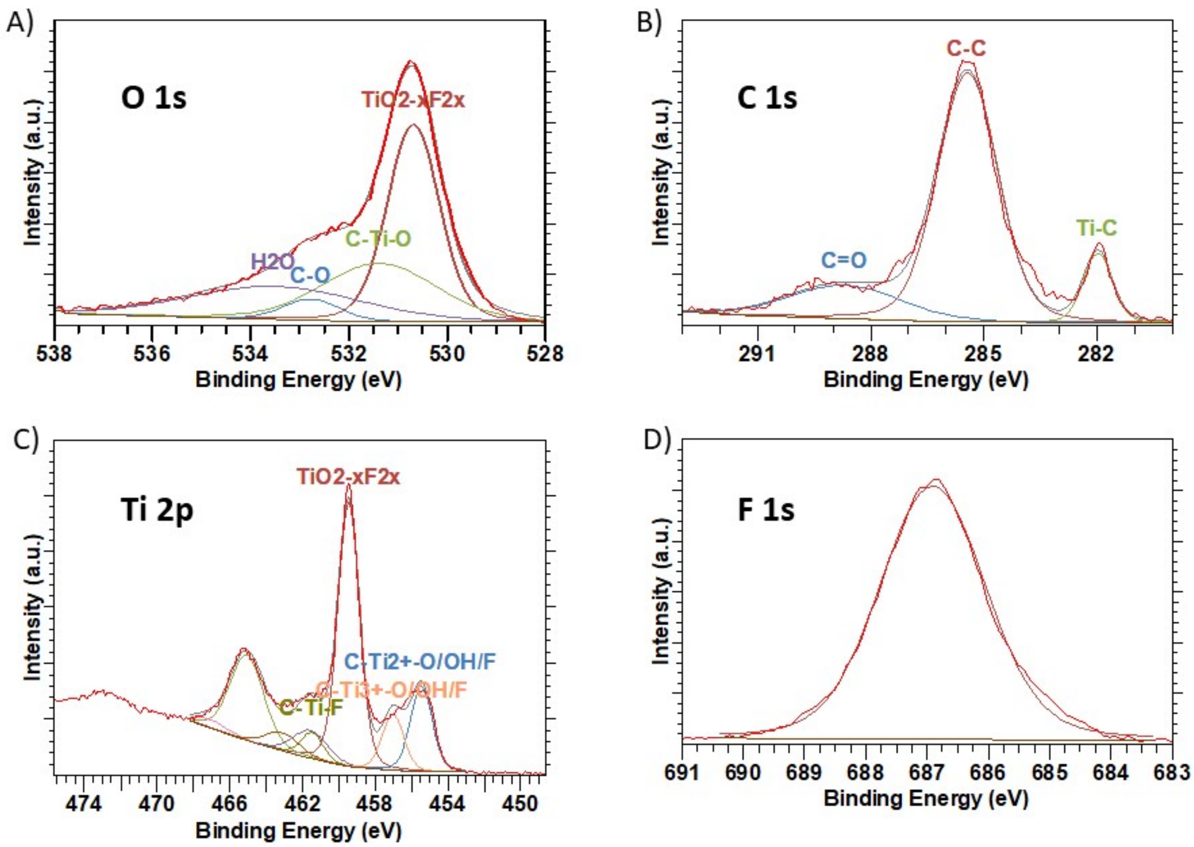

Figure S5 XPS component peak fittings for the batch sample: A) O 1s, B) C 1s, C) Ti 2p and D) F 1s.

For the O 1s, four peaks are fitted. The peak at 530.68 eV is assigned to $TiO_{2-x}F_{2x}$, following Benchakar et al. [7] and Natu et al [5]. The peak at 531.38 eV is assigned to C-Ti-O [5] while the peak at 532.77 eV is assigned to C-O bonds [8]. The peak at 533.51 eV is assigned to adsorbed water [5]. We note the high percentage (43.16%) that corresponds to oxides and oxyfluorides, which indicates oxidation of the sample. The C 1s spectra was fitted with three peaks. The peak at 281.97 eV is the calibration peak, and it was attributed to C-Ti-C bonds [5]. The 285.43 eV peak is the adventitious carbon peak (C-C) and the peak at 288.69 eV was assigned to double-bonded carbon and oxygen, according to Schultz et al. [8] . We note the high percentage of adventitious carbon (72.36%) on this sample. The Ti 2p region is fitted with 4 peaks. The peaks at 455.47 eV and 457.02 eV are attributed to C-Ti^{2+}/O/OH/F and C-Ti^{3+}/O/OH/F, respectively [7]. The peak at 459.46 eV is attributed to $TiO_{2-x}F_{2x}$ [5, 7] and the peak at 461.47 eV is assigned to C-Ti-F bonds [9]. The Ti 2p peak fitting also seem to indicate a high degree of oxidation of the sample (61.19% for $TiO_{2x}F_{2x}$). The F 1s region is fitted with a single peak at 686.91 eV, which seems to indicate mostly F impurities such as AlF_3 or $Ti_2O_{2-x}F_{2x}$ in the sample [9].

Table S3 XPS peak fittings for the batch sample

Region	Binding energy (eV)	FWHM	Percentage (%)	Assigned to
O 1s	530.68	1.25	43.16	$TiO_{2-x}F_{2x}$
	531.38	2.76	27.96	C-Ti-O
	532.77	1.35	4.95	C-O
	533.51	4.17	23.92	H_2O
C 1s	281.97	0.91	9.78	C-Ti-C
	285.43	1.89	72.36	C-C
	288.69	3.48	17.86	C=O
Ti $2p_{3/2}$ ($2p_{1/2}$)	455.47 (461.57)	1.41 (2.19)	19.84	C-Ti^{2+}/O/OH/F
	457.02 (463.12)	1.41 (2.19)	12.93	C-Ti^{3+}/O/OH/F
	459.46 (465.06)	1.41 (2.19)	61.19	$TiO_{2-x}F_{2x}$

	461.47 (467.07)	1.41 (2.19)	6.04	C-Ti-F
F 1s	686.91	2.04	100	F impurities

The batch sample also had two additional peaks, for Al and Cl. The figures and fitting data for these are below in Fig. S6 and Table S4.

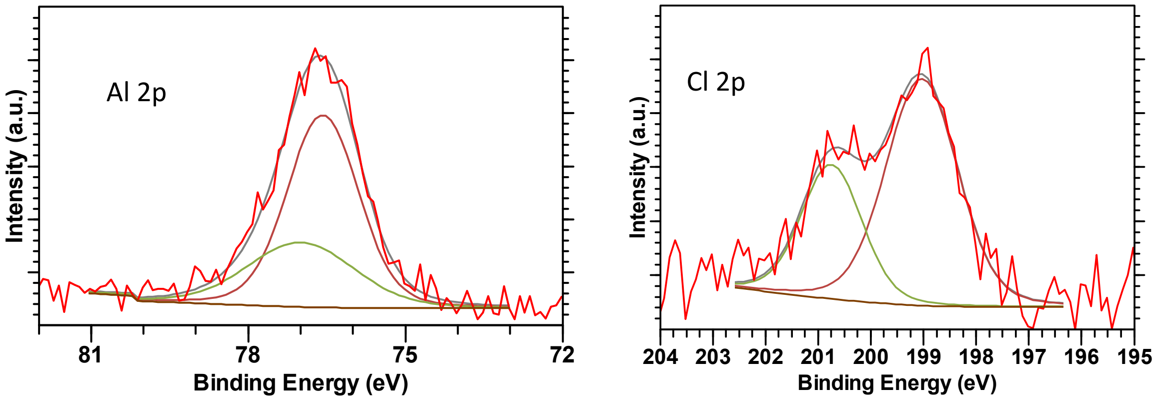

Figure S6 XPS component peak fittings for the batch sample. Al 2p (left-hand side) and Cl 2p (right-hand side).

Table S4 XPS peak fittings for the Al 2p and Cl 2p peaks in the batch sample

Region	Binding energy (eV)	FWHM	Fraction
Al $2p_{3/2}$	76.58	1.61	0.66
Al $2p_{1/2}$	77.00	2.40	0.33
Cl $2p_{3/2}$	199.03	1.61	0.66
Cl $2p_{1/2}$	200.75	1.36	0.33

The doublet splitting in the Al was fixed at 0.42 eV, as observed in the literature [10]. The doublet splitting for Cl was not fixed and the obtained value (1.72 eV) is close to the one from literature, which is 1.60 eV [10].

Continuous sample

The XPS fittings for the O 1s, C 1s, Ti 2p and F 1s regions are shown below in Figure S7. The peak fitting data is displayed in Table S5.

Much of the fitting procedure and peak assignment adopted for the batch sample is followed for the continuous sample. For the O 1s, four peaks are fitted. The peak at 530.77 eV is assigned to $TiO_{2-x}F_{2x}$ [5, 7]. The peak at 531.8 eV is assigned to C-Ti-O [5] while the peak at 532.64 eV is assigned to C-O bonds [8]. The peak at 533.81 eV is assigned to adsorbed water [5]. The oxides and oxyfluorides constitute most of the peak (64.49%), which seems to indicate a high degree of oxidation in the samples. However, it should be noted that XPS is a surface technique and the degree of oxidation noted here is not observed in any of the other characterisation techniques. The C 1s spectra was fitted with three peaks. The peak at 281.99 eV is the calibration peak, and it was attributed to C- Ti-C bonds [5]. The 285.43 eV peak is the adventitious carbon peak (C-C) and in a comparable way to the batch sample, the peak at 288.69 eV was assigned to double-bonded carbon and oxygen [8]. There is again a high percentage of adventitious carbon (69.79%) on the sample. The Ti 2p region is fitted with 4 peaks. The peaks at 455.61 eV and 457.16 eV are attributed to C-Ti^{2+}/O/OH/F and C-Ti^{3+}/O/OH/F, respectively [7]. The peak at 459.51 eV is attributed to $TiO_{2-x}F_{2x}$ [5, 7] and the peak at 461.60 eV is assigned to C-Ti-F bonds [9], as done for the batch sample. The peak fitting for the Ti 2p also seems to indicate a large degree of oxidation in the sample (51.29%). The F 1s region is fitted with a single peak at 685.80 eV, which is again assigned to F impurities such as AlF_3 or $Ti_2O_{2-x}F_{2x}$ [9].

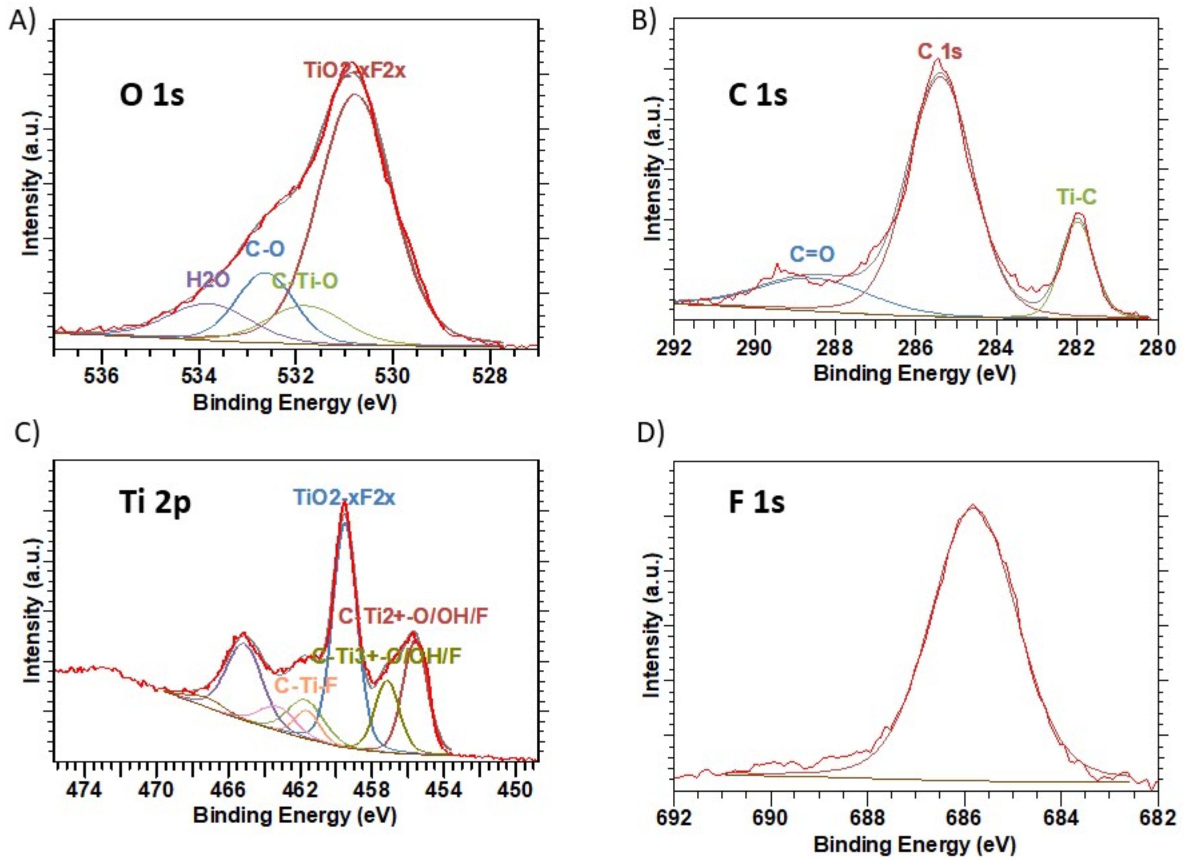

Figure S7 XPS component peak fittings for the continuous sample: A) O 1s, B) C 1s, C) Ti 2p and D) F 1s.

Table S5 XPS peak fittings for the continuous sample

Region	Binding energy (eV)	FWHM	Percentage (%)	Assigned to
O 1s	530.77	1.80	64.49	$TiO_{2-x}F_{2x}$
	531.80	1.84	11.15	C-Ti-O
	532.64	1.41	14.11	C-O
	533.81	2.01	10.25	H_2O
C 1s	281.99	0.96	14.23	C-Ti-C
	285.39	1.91	69.79	C-C
	288.53	3.18	15.98	C=O
Ti $2p_{3/2}$ ($2p_{1/2}$)	455.61 (461.71)	1.61 (2.37)	25.85	C-Ti^{2+}/O/OH/F
	457.16 (463.26)	1.61 (2.37)	16.41	C-Ti^{3+}/O/OH/F
	459.51 (465.11)	1.61 (2.37)	51.29	$TiO_{2-x}F_{2x}$
	461.60 (467.20)	1.61 (2.37)	6.45	C-Ti-F
F 1s	685.80	2.10	100	F impurities

The continuous sample also had two peaks, for Ca and Cl, which are shown and analysed below in Figure S8.

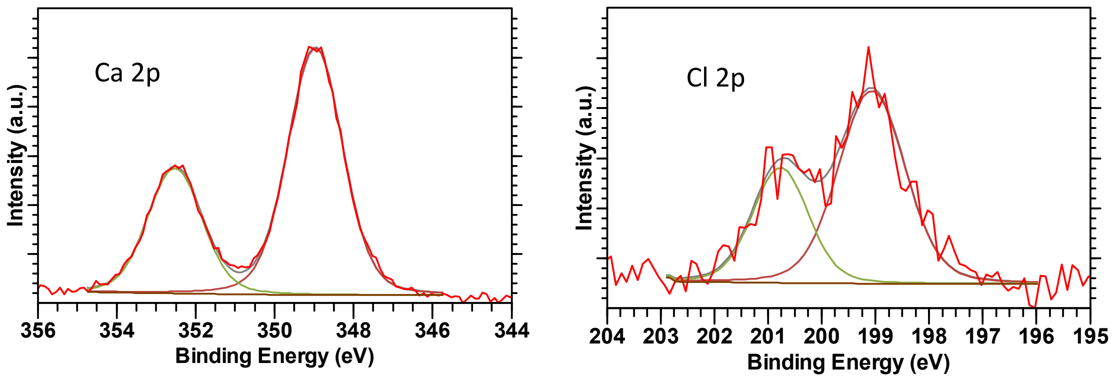

Figure S8 XPS component peak fittings for the continuous sample. Ca 2p (left-hand side) and Cl 2p (right-hand side).

Table S6 XPS peak fittings for the Ca 2p and Cl 2p peaks in the continuous sample

Region	Binding energy (eV)	FWHM	Fraction
Ca $2p_{3/2}$	348.97	1.64	0.66
Ca $2p_{1/2}$	352.53	1.62	0.33
Cl $2p_{3/2}$	199.07	1.46	0.66
Cl $2p_{1/2}$	200.77	1.22	0.33

We note that the peak separation in the Ca is consistent with literature values (3.5 eV) [10] and that the Cl peak separation is again within the values reported in literature (1.60 eV) [10].

As noted in the literature, XPS peak fitting in MXenes can be marred with inconsistencies and analysis is difficult for most samples [5]. Nonetheless, our analysis seems to indicate that:

1. Al is only present in the batch sample, which shows that the continuous sample is fully etched and that the washing procedure in the continuous reactor removes all the Al.
2. There is presence of Cl in both samples from the etchant solution (HCl).
3. There is some ambiguity in the results with respect to the oxidation of samples. The O 1s data seem to indicate a higher degree of oxidation in the continuous sample (64.49% vs 43.16%) while the Ti 2p data indicates otherwise (51.29% in the continuous sample vs 61.19% in the batch sample). In both cases and given limited contact with the atmosphere, the oxidation is probably due to overetching, which indicates that experiments are running longer than needed to etch Al. However, as noted above, XPS is a surface technique which is highly sensitive to even small traces of oxidation on the surface of the material.
4. There is Ca^{2+} contamination in the continuous sample, as also seen in the EDS analysis.
5. There is more water in the batch sample than in the continuous sample, which might be due to different drying of the samples.

NMR Analysis

Table S7 NMR peak fitting for the ^{1}H SS NMR for the batch sample

Amplitude	Position	Line Width	Ratio
4.548	8.082	1.469	0.370
3.571	6.100	0.909	0.280
19.980	5.134	0.743	0.881

Table S8 NMR peak fitting for the ^{1}H SS NMR for the continuous sample

Region	Binding energy (eV)	FWHM	Fraction
10.730	9.000	29.857	0.0153
2.124	8.423	2.008	$9.03E^{-07}$

5.170	5.800	1.298	0.114
21.887	4.662	0.902	0.786
2.370	2.497	1.595	0.001
0.164	0.499	0.399	0.940

TEM

Transmission electron microscopy was performed on the samples obtained through the batch method and the semi-continuous method to investigate differences in morphology. Fig S9 shows the imaging done on flakes of both samples, showing minor differences between the samples. The only salient point is the fact that flakes from the semi-continuous method seem slightly more delaminated (hence, more transparent to the electron beam) than the samples obtained from the batch method.

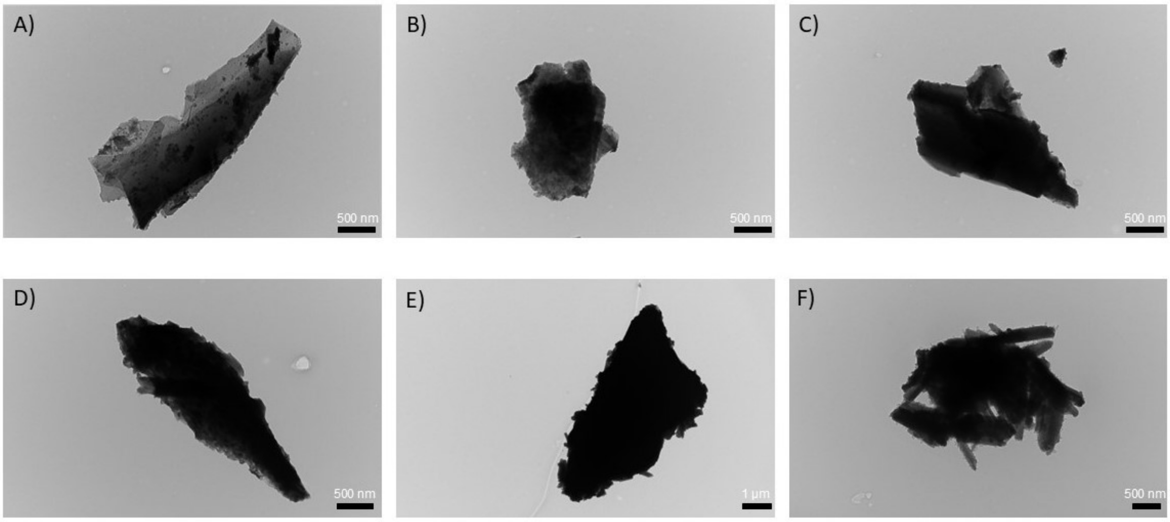

Figure S9 Transmission electron microscopy imaging of the batch and semicontinuous MXene samples. A, B and C are different flakes of the semi-continuous sample, all imaged with a magnification of 26500 x. D, E and F are different flakes of the batch sample imaged with a magnification of 26500 x, 9900 x, and 20500x, respectively.

UV-Visible spectroscopy

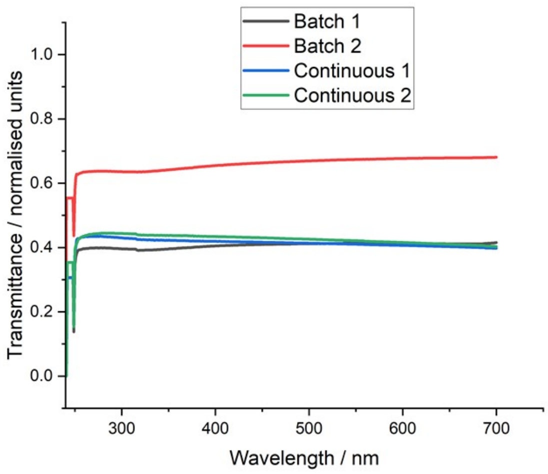

Figure S10 UV-Visible spectroscopy done on two sets of samples obtained from the batch and continuous method.

References

1. Clark, M.J., et al., *3D printed filtration and separation devices with integrated membranes and no post-printing assembly.* Reaction Chemistry & Engineering, 2024. **9**(2): p. 251-259.
2. Price, A.J.N., et al., *An open source toolkit for 3D printed fluidics.* Journal of Flow Chemistry, 2021. **11**(1): p. 37-51.
3. Maughan, P.A., et al., *In Situ Investigation of Expansion during the Lithiation of Pillared MXenes with Ultralarge Interlayer Distance.* Journal of Physical Chemistry C, 2021. **125**(38): p. 20791-20797.
4. Ghidiu, M., et al., *Conductive two-dimensional titanium carbide 'clay' with high volumetric capacitance.* Nature, 2014. **516**(7529): p. 78-U171.
5. Natu, V., et al., *A critical analysis of the X-ray photoelectron spectra of Ti3C2Tz MXenes.* Matter, 2021. **4**(4): p. 1224-1251.
6. Fairley, N., et al., *Systematic and collaborative approach to problem solving using X-ray photoelectron spectroscopy.* Applied Surface Science Advances, 2021. **5**: p. 100112.
7. Benchakar, M., et al., *One MAX phase, different MXenes: A guideline to understand the crucial role of etching conditions on Ti3C2Tx surface chemistry.* Applied Surface Science, 2020. **530**: p. 147209.
8. Schultz, T., et al., *Surface Termination Dependent Work Function and Electronic Properties of Ti3C2Tx MXene.* Chemistry of Materials, 2019. **31**(17): p. 6590-6597.
9. Halim, J., et al., *X-ray photoelectron spectroscopy of select multi-layered transition metal carbides (MXenes).* Applied Surface Science, 2016. **362**: p. 406-417.
10. NIST, *NIST X-ray Photoelectron Spectroscopy Database (SRD 20), Version 5.0*.
